# Evidence implicating the commensal microbiota in shaping anti-tumor immunity in melanoma

**DOI:** 10.1186/2051-1426-2-S3-O11

**Published:** 2014-11-06

**Authors:** Ayelet Sivan, Leticia Corrales, Thomas Gajewski

**Affiliations:** 1 University of Chicago, Chicago, IL, USA

## 

T cell infiltration of solid tumors is found in a subset of cancer patients and is associated with favorable patient outcomes. This demonstrates the capacity of the immune system to mount protective anti-tumor responses in some cancer patients, yet the underlying mechanisms mediating the presence or absence of a T cell infiltrate are not well understood. Individuals exhibit remarkable diversity in their commensal microbiota, which in turn influences systemic immune responses, yet the role of microbiota in shaping spontaneous immunity against tumors is not known. Here we sought to examine the effect of microbial composition on the immune response to melanoma by comparing subcutaneous B16 melanoma growth in genetically identical C57BL/6 mice derived from two different facilities, Jackson laboratories and Taconic farms, which have been shown to differ in their commensal microbes. We found that B16.SIY tumors exhibited an increased growth rate in mice derived from Taconic (p < 0.02), which was associated with reduced induction of tumor-specific IFN-γ-producing CD8^+ ^T cells (p < 0.009). A markedly increased number of SIY antigen-specific CD8^+ ^T cells was found to accumulate within the tumor microenvironment in Jackson versus Taconic mice (p < 0.03). Strikingly, the differences in tumor growth, endogenous T cell priming, and SIY antigen-specific T cell infiltration were ablated in animals cohoused for at least two weeks prior to injection of tumor cells, consistent with a microbiota-derived effect. We thus asked whether transfer of Jackson fecal material into Taconic mice by oral gavage prior to tumor inoculation was sufficient to confer protective anti-tumor responses. Indeed, Taconic mice that received Jackson feces exhibit dramatically slower tumor growth (p < 0.015) and increased numbers of SIY antigen-specific CD8^+ ^T cells at the tumor site, compared to Taconic mice that received Taconic feces. To pursue mechanisms that might explain these differences, we transferred CFSE-labeled SIY-specific 2C TCR Tg T cells into tumor-bearing Jackson and Taconic mice and tested their proliferation and acquisition of IFN-γ production ex vivo. 2C T cells responding to tumors in Taconic mice produced significantly less IFN-γ than 2C T cells responding to tumors in Jackson mice, likely pointing to a difference at the level of antigen-presenting cells. No difference in Th17 induction was observed. Our data provide evidence implicating the microbiota in shaping anti-tumor immunity in a pre-clinical model of melanoma. Gaining further understanding of the mechanisms underlying this effect may provide the foundation for rational manipulation of commensal microbes as a cancer therapeutic.

